# Decades of Discovery: Contributions of the Atherosclerosis Risk in Communities (ARIC) Study to Dementia Research

**DOI:** 10.1002/alz70860_097400

**Published:** 2025-12-23

**Authors:** Rebecca F. Gottesman, Josef Coresh, David S. Knopman, Pamela L. Lutsey, Priya Palta, A. Richey Sharrett, Lynne E Wagenknecht, Gwen Gwen Windham, Thomas H. Mosley

**Affiliations:** ^1^ National Institute of Neurological Disorders & Stroke, Bethesda, MD, USA; ^2^ Departments of Population Health and Medicine, New York University Grossman School of Medicine, New York, NY, USA; ^3^ Mayo Clinic, Rochester, MN, USA; ^4^ University of Minnesota School of Public Health, Minneapolis, MN, USA; ^5^ University of North Carolina at Chapel Hill, Chapel Hill, NC, USA; ^6^ Johns Hopkins University Bloomberg School of Public Health, Baltimore, MD, USA; ^7^ Wake Forest University School of Medicine, Winston‐Salem, NC, USA; ^8^ University of Mississippi Medical Center, Jackson, MS, USA; ^9^ University of Mississippi Medical Center, The MIND Center, Jackson, MS, USA

## Abstract

The Atherosclerosis Risk in Communities (ARIC) study started in 1987‐1989 (visit 1), when participants from four U.S. communities were 45‐64yo. Participants have been seen at up to 10 additional in‐person visits with annual/semi‐annual telephone calls to ascertain clinical events, hospitalizations, and other health outcomes. In 2011‐2013, at ARIC visit 5, the ARIC Neurocognitive Study (ARIC‐NCS) was initiated for surviving participants, incorporating neuropsychological assessments at multiple in‐person visits and informant interviews to support cognitive adjudication. A subset of participants have undergone brain MRI with measurement of brain volumes, cortical thickness, arterial disease and vascular lesions, with a smaller subset having brain florbetapir PET. Blood‐based Alzheimer's disease (AD) biomarkers are available at multiple visits along with extensive measurement of vascular risk factors, vascular markers, and ‐omic (e.g.genomic and proteomic) data. This presentation will focus on the available data in ARIC, the methods underlying its collection and subsequent analysis, and some of the primary contributions of this study to the field. With 37 years of follow‐up, a great strength of ARIC is the ability to evaluate risk factors in midlife; ARIC studies have supported an association of midlife hypertension, diabetes, physical inactivity, and other vascular risk factors with reduced dementia risk, even among those with increased genetic risk for dementia. ARIC studies have demonstrated associations between stroke and dementia, and have linked vascular risk factors with blood‐based and imaging biomarkers of AD‐related dementias. ARIC has demonstrated the importance of hearing loss in dementia risk, and a subset of ARIC participants were recruited into (and were those who most benefited from the intervention of) the ACHIEVE study of hearing aid use for reduction of dementia. Proteomic studies have identified markers of dementia risk when evaluated in midlife. Recently, ARIC data informed updated estimates on lifetime risk of dementia, where it was estimated that among individuals surviving beyond age 55, 42% would develop dementia over their lifetime through age 95. ARIC studies continue to focus on risk factors for dementia, particularly now in the oldest old, and on contributors to cognitive reserve and resilience. ARIC follows a model of data sharing and broad collaboration.

